# Supplementary material for: Shotgun proteomic profiling of dormant, ‘non-culturable’ Mycobacterium tuberculosis
Source: PLoS One. 2022 Aug 9;17(8):e0269847. doi: 10.1371/journal.pone.0269847 (PMC9362914; doi:10.1371/journal.pone.0269847)
Supplement: Attachment — Submitted filename: Responce to Reviewers.docx [file pone.0269847.s002.docx]

**Shotgun proteomic profiling of dormant, ‘non-culturable’ *Mycobacterium tuberculosis***

***Reviewer 1:***

“*This work describes the proteins / antigen that are differentially regulated during dormancy. The study was carried out systematically and is more significant to enrich the current knowledge of the biochemical pathway and immunogenic altercations during Mtb dormancy. The authors have extensively generated knowledge in the post persistence state of the pathogen, however the following needs to be addressed.*

1. *Among the various hypothetical invitro models proposed by various researchers in this field related to non-replicating persisters, some of them were extensively studied for their closeness to in vivo conditions through evidences that might be analogous to the chain of events occurring while the pathogen is proceeding to a state of persistence invivo. The authors need to provide such evidences for this particular model of self-acidification, so that consequential elucidation of the events leading to knowledge generated reveal the closeness to those events occurring invivo. It is difficult to find such cross references for this particular model which might be significant to readers to assimilate possible post latency mechanistic consequences described in the manuscript*.”

- We have added a detailed description in Introduction section why our model is applicable for modeling of latent TB in vivo.

«2. *Time-point of CFU and MPN was not clearly specified in the methods section. Also state whether the absence of contamination was ensured periodically*.»

- Added, Lines 186 (section “Evaluation of cell viability by CFU and MPN assays”) & 342 ( section “Dormant mycobacterial cells subjected to the proteomic analysis and the results of proteomic profiling”) in the revised manuscript.

«3. *The study was carried out in laboratory strain. It would have been better if the same was done in the clinical strain with different treatment outcomes*.»

- We agree, it could be interesting to use a clinical strain. At the same time, the most important publications on the subject from different laboratories were conducted on the H37Rv strain - what makes its crucial for the comparison of the results obtained in the current study with the previously published results.

***Reviewer 2:***

«*In this study, the authors have identified differential proteome profile between actively growing and dormant Mycobacterium tuberculosis. This is the continuation of their previous study where they found diverse protein production in non culturable cells. I found the study very informative and well presented. However, I would suggest to provide the information from section "3.3 Differential analysis" in the tabular form as well*.»

- The results of statistical significance can be found in the supplementary excel file: “S1. Table. The results of proteomic LC-MS analysis of *M. tuberculosis* H37Rv in dormancy and activity” in the datasheet “The results of differential analysis”.

The corresponding indices were similarly added for the sections “Annotation of immunogenic proteins accumulated in dormant cells”

and “Annotation of proteins accumulated in dormancy – potential prodrug activators” as well – where the lists of antigenic and enzymatic proteins are discussed.
